# Second Generation Small Pixel Technology Using Hybrid Bond Stacking †

**DOI:** 10.3390/s18020667

**Published:** 2018-02-24

**Authors:** Vincent C. Venezia, Alan Chih-Wei Hsiung, Wu-Zang Yang, Yuying Zhang, Cheng Zhao, Zhiqiang Lin, Lindsay A. Grant

**Affiliations:** OmniVision Technologies, Inc., Santa Clara, CA 95054, USA; alan.hsiung@ovt.com (A.C.-W.H.); wz_yang@ovt.com (W.-Z.Y.); yuying.zhang@ovt.com (Y.Z.); cheng.zhao@ovt.com (C.Z.); zlin@ovt.com (Z.L.); lindsay.grant@ovt.com (L.A.G.)

**Keywords:** CIS, BSI, stacked, hybrid-bond, 1.0 µm, 0.9 µm, NIR

## Abstract

In this work, OmniVision’s second generation (Gen2) of small-pixel BSI stacking technologies is reviewed. The key features of this technology are hybrid-bond stacking, deeper back-side, deep-trench isolation, new back-side composite metal-oxide grid, and improved gate oxide quality. This Gen2 technology achieves state-of-the-art low-light image-sensor performance for 1.1, 1.0, and 0.9 µm pixel products. Additional improvements on this technology include less than 100 ppm white-pixel process and a high near-infrared (NIR) QE technology.

## 1. Introduction

**Figure 1:**
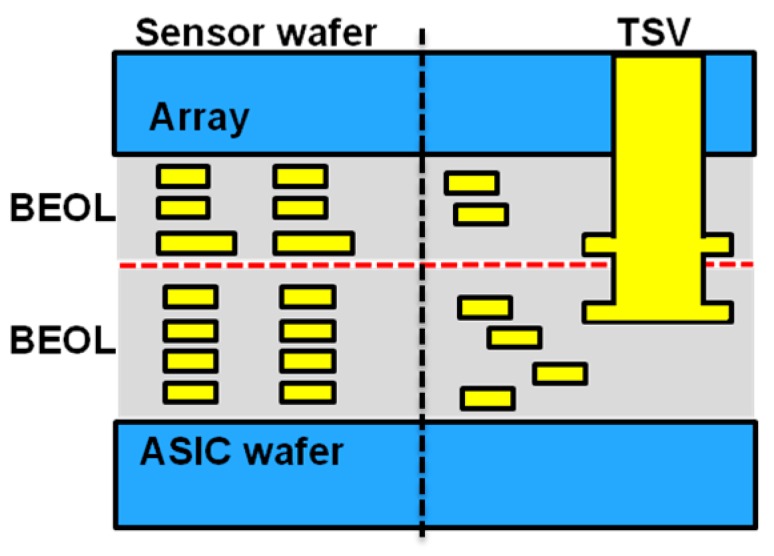
First-generation BSI-CIS stacking with oxide-oxide bonding and TSV.

**Figure 2:**
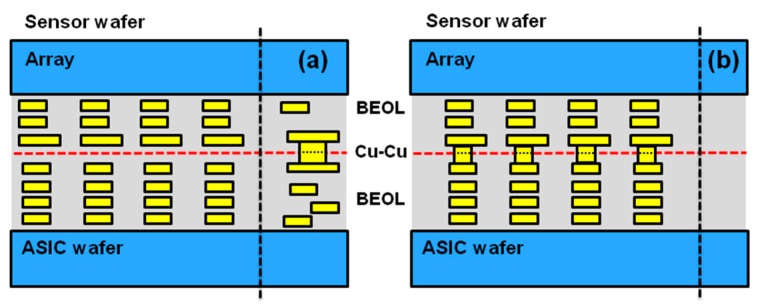
Hybrid bond-stacking schematic, showing the Cu-Cu interconnects outside the array (**a**) and within the array (**b**).

The first generation of back-side illuminated (BSI) stacked products used oxide bonding to physically bond wafers, and through-silicon vias (TSV) to form electrical connections [1,2]. In addition to the chip size reduction offered by wafer stacking, improvements in photo-diode performance are possible with “sensor only”-type processing that would negatively impact ASIC performance on non-stack BSI or even front-side illuminated sensors, such as new materials or thermal conditions [1]. In our second generation of stacking technologies, wafers are connected by the top-metal layer and the inter-layer dielectrics of the sensor and ASIC wafer, forming a hybrid oxide-oxide and metal-metal bond that physically and electrically connects the two wafers simultaneously. Hybrid bonding offers significant improvements in design and layout flexibility over the TSV stacking which consumes chip space to create deep vias and are aspect-ratio pitch limited; for examples, see Figure 1 and Figure 2. While TSVs can only be placed outside the array, hybrid-bond connections can be made both outside (Figure 2a) and inside the array (Figure 2b). In addition, hybrid bonding connections do not interfere with sensor or circuit performance, as is the case with the TSV Keep-Out Zone (KOZ) [3]. Using the flexibility hybrid bonding offers, the overall chip size of OmniVision’s 1 um, 16 MP stacked-chip product was reduced by ~10% [4].

**Figure 3:**
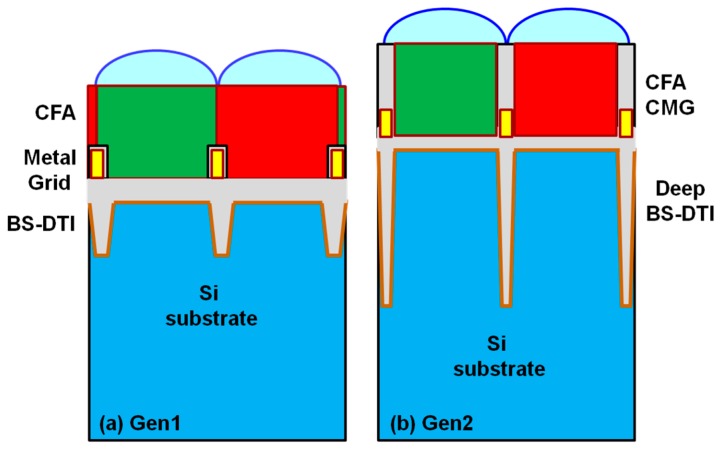
Gen1 (**a**) and Gen2 (**b**) schematic; Gen2 BSI stack has thicker silicon, narrower and deeper BS-DTI, and a composite metal-oxide, back-side grid.

Back-side, deep-trench isolation (BS-DTI), as well as a buried color filter array (CFA), were implemented in OmniVision’s first generation of stacking products to improve image quality [1]; BS-DTI to control electrical and optical crosstalk, and the buried CFA to reduce the optical stack height. Figure 3 is a cross section diagram comparing the Gen1 and Gen2 structures. In the Gen2 technology, the silicon thickness and the BS-DTI depth are increased to improve sensitivity while controlling crosstalk. A new composite oxide-metal grid (CMG) is introduced, in which an additional oxide-grid structure is added above the metal grid, separating neighboring color filters. As will be shown below, the Gen 2 pixel structure and process improves sensitivity without degrading crosstalk to achieve excellent low-light performance.

## 2. Results and Discussion

**Figure 4:**
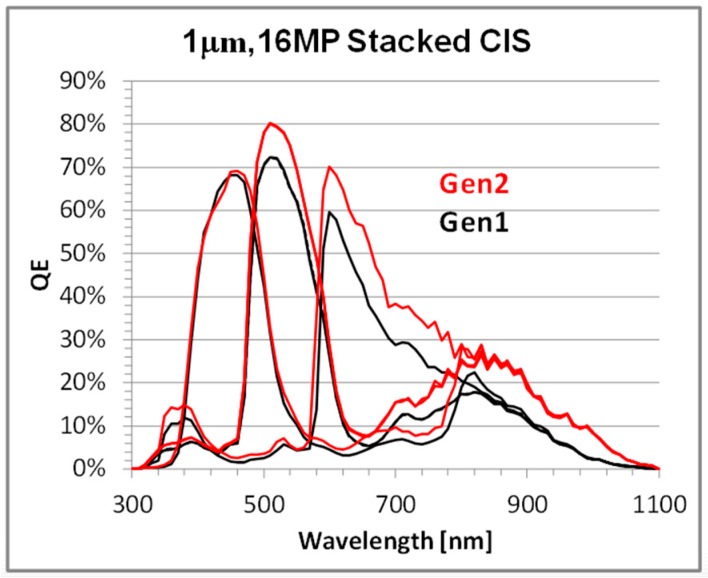
QE comparing Gen1 and Gen2 1 µm, 16 MP technologies.

**Figure 5:**
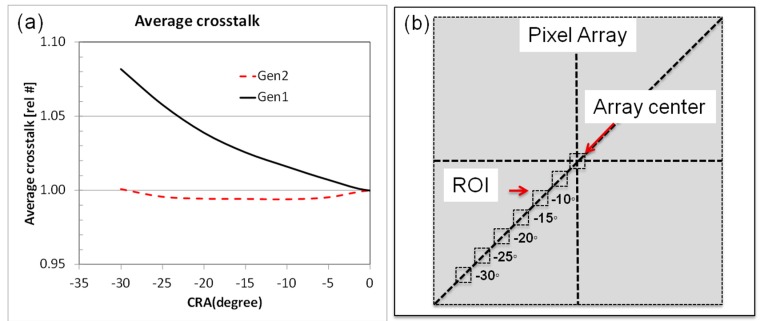
(**a**) Crosstalk vs angle, calculated from QE curves measured in a fix ROI along a diagonal as shown in the (**b**). Angles are the CRA of the module lens at each ROI.

**Figure 6:**
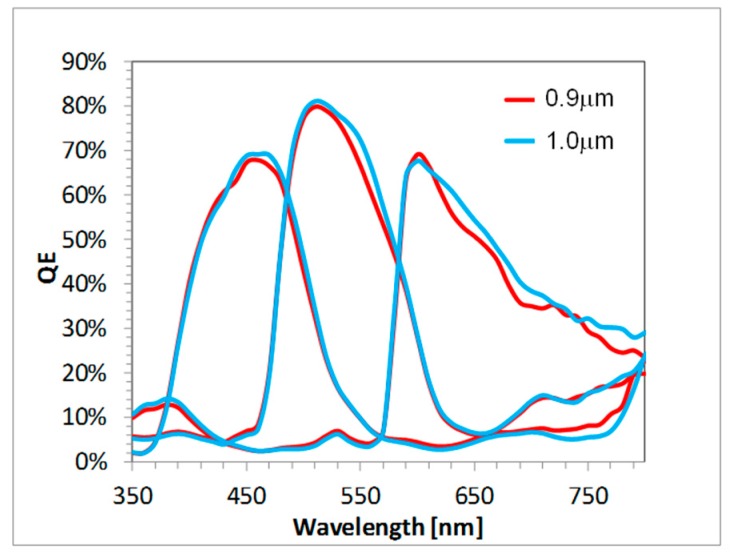
The QE from 1 µm and 0.9 µm pixel products, both using the Gen2 BSI-stacking technology.

The quantum efficiency (QE) from 1 um, 16 MP Gen1 and Gen2 products is compared in Figure 4. The peak green and red QE are increased 10% and 15%, respectively, while the NIR QE (850 nm) is increased by ~50%. The Gen2 angular response improvement was reported in [4]. Figure 5a compares the Gen1 and Gen2 average crosstalk along the chief ray angle (CRA) of the sensor; crosstalk is calculated from QE measurements along a diagonal line from array center to edge, where the angle is determined by the CRA of the modules lens (Figure 5b). The crosstalk increases at large angles on a Gen1 sensor, while it remains relatively constant on a Gen2 stacked sensor. Figure 6 compares the QE from a 1.0 µm pixel CIS product to that from a 0.9 µm pixel CIS product, both using the Gen2 stacking technology described above. Despite the decrease in pixel size, the 0.9 µm pixel has the same high QE and low crosstalk as the 1.0 µm pixel, demonstrating that the Gen2 optical benefits can be extended to sub 1 µm pixel pitches.

**Figure 7:**
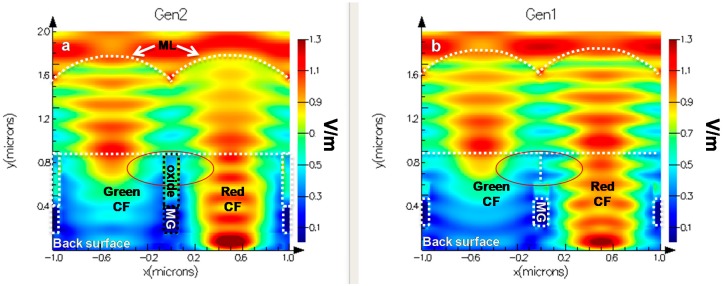
FDTD simulation comparing the light confinement of Gen2 (**left**) and Gen1 (**right**). Simulations used a monochromatic plan wave at, 630 nm TE mode, incident on the Green and Red pixel of each structure. Structure features are labeled and highlighted.

Finite-difference time-domain (FDTD) simulations were used to understand the Gen2 improvement in optical performance. Figure 7 shows the simulation results comparing the Gen2 and Gen1 optical stacks. In the simulation, a 630 nm, monochromatic plane wave (Transverse electric mode (TM)) is used to illuminate neighboring pixels in each structure; the left pixel has a green color filter while the right pixel has a red color filter. The only difference between the pixels in each simulation is the color filter index of refraction. The figure plots the electric field propagation through the micro lens (ML) and color filter. The Gen1 and Gen2 structures are outlined by dashed lines and labeled for clarity. The main difference between structures is the extra silicon dioxide barrier between the color filters in the Gen2 stack. The silicon-dioxide refractive index (~1.45) is lower than that of the CF index (~1.6–2.0), resulting in light reflection at the CF/oxide interface. In the case of Gen1, partially filtered light can pass between color filters, resulting in signal loss due to optical crosstalk. The crosstalk difference is seen in the Figure 7 simulation results; comparing the circled areas, the electric field propagation between color filters is eliminated by the Gen2 CMG. The better light confinement in the CF by the CMG results in higher QE and lower optical crosstalk, which is consistent with the QE data in Figure 4.

**Figure 8:**
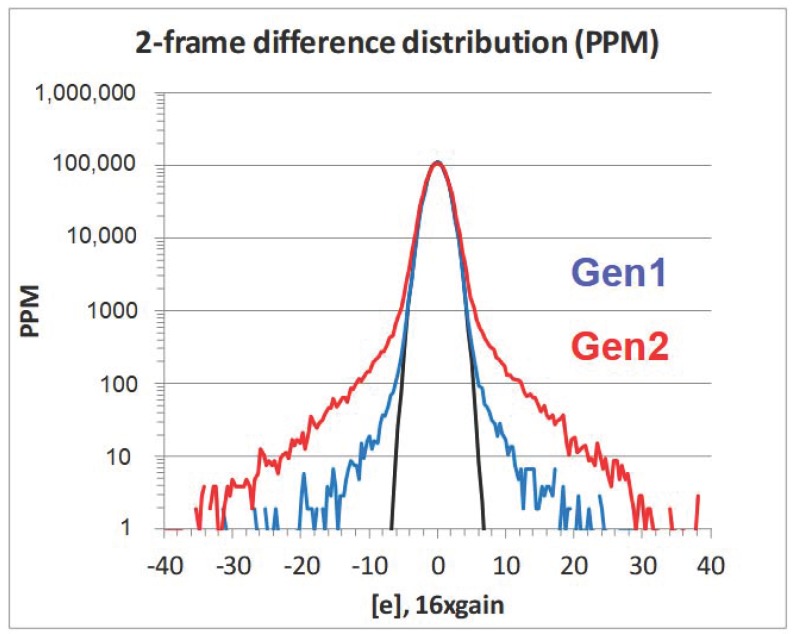
Two-frame difference image, noise histogram comparing the 1 µm, 16 MP images sensors that use the Gen1 and Gen2 technology.

**Figure 9:**
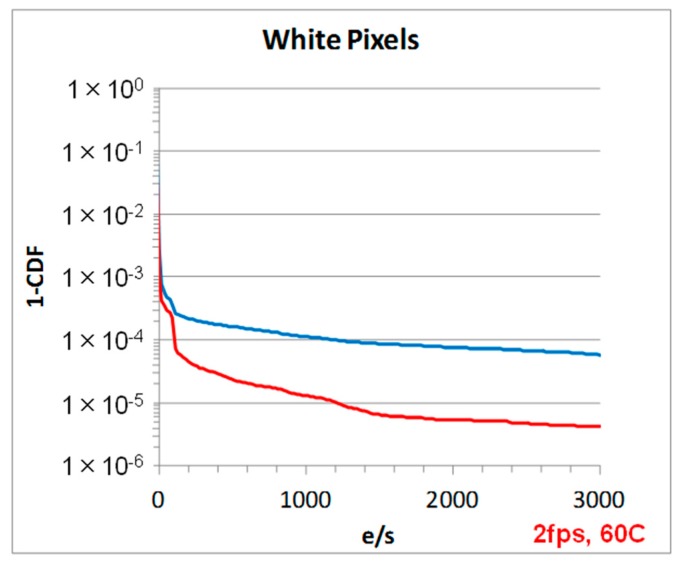
Plot of 1-cumulative density function (1-CDF) comparing the dark image from low white-pixel, PD process. 1 µm, 16 MP (2 fps, 60 C).

In addition to the optical performance benefits, the Gen2 technology noise, white pixel (WP) and dark current performance has been improved over the Gen1, see Table 1. Figure 8 is a plot of the noise histogram from a two-frame difference image showing a ~2x improvement in the RTS noise. The low level of noise was achieved by maximizing the source follow area [5] and reducing source follower traps by minimizing etching damage, reducing dangling bonds, and by device channel engineering as discussed in [6]. The WP defect improvement is shown in Figure 9. A greater than 2x reduction in WP is achieved by engineering the photo-diode implant process to reduce any high electricity field regions and suppress the photo-diode (PD) depletion regions from interacting with high defect surfaces, such as front, back, and BS-DTI surfaces. The back surface passivation, for both Gen1 and Gen2, is achieved by deposition of a negatively charged oxide film [7]. In the Gen2 technology, the negative charge density in this film is increased, resulting in an improved dark current, as shown in Table 1.

The improved angular performance, as discussed in [4] and shown in Figure 5, is due to the BS-DTI. A relatively shallow BS-DTI was introduced in the Gen1 technology [1] and resulted in improvement in the crosstalk versus angle performance. This benefit was extended in Gen2 by increasing the DTI depth by ~3x, resulting in the elimination of the crosstalk at high angles (Figure 5).

**Figure 10:**
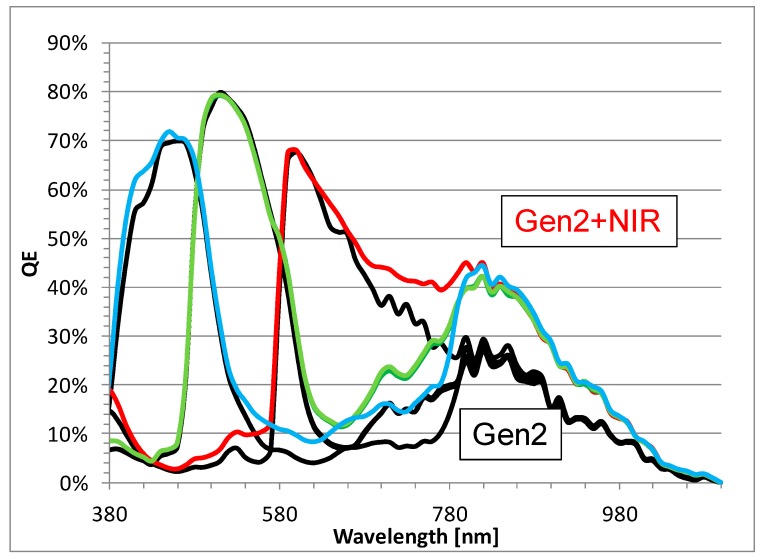
QE comparison from a 1 µm, 16MP Gen2 image sensor to one with an additional process for NIR enhancement.

It is well known that increasing the silicon thickness increases the long-wavelength QE [8]. As discussed above, the Gen2 thickness is increased, thereby increasing the NIR QE. Unlike the TSV-bonding, which is limited by the etching and filling of deep trenches, the hybrid bond technology is more flexible for increasing the final silicon thickness. In addition to increasing the BSI thickness, we improve the NIR QE with a new technology [9] to achieve state-of-the-art NIR performance for 1 µm pixels. Figure 10 shows the QE curve from a 1 µm pixel NIR performance. The QE at 850 nm and 940 nm increases to 40% and 20% respectively, without any significant impact on the visible QE.

## 3. Conclusions

**Figure 11:**
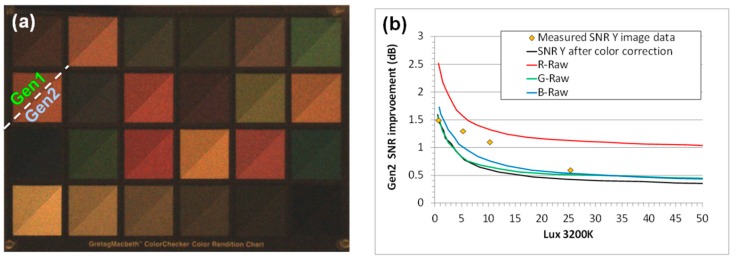
(**a**) Combined Macbeth chart images from 16 MP, 1 µm pixel Gen1 and Gen2 products to show the Gen2 low light (5 Lux) sensitivity improvement. (**b**) Gen2 SNR improvement vs. lux level, including measured data and simulated results (F2.0, 15 fps).

The improvements in the Gen2 over the Gen1 technology are highlighted in Table 1. The higher sensitivity and better SNR10 [10] is achieved by increasing the BSI silicon thickness, extending the BS-DTI deeper, and introducing the CMG. These optical benefits are maintained at sub 1 µm pixels, such as 0.9 µm. The lower noise is due to an improved source follower design and process, while the dark current improvement is achieved by photo-diode and surface passivation engineering. The higher QE and low crosstalk, as well as the improved noise, results in excellent low-light performance, as is shown in Figure 11, where Macbeth chart images taken by a Gen2 and Gen1 CIS sensor at 5 Lux are overlaid for comparison. Figure 11b plots the simulated and measured improvement in SNR vs lux level for a 1 µm Gen2 CIS product. The SNR improvement is most significant at low lux levels. The Gen2 technology is currently in mass production for 1.1 µm, 1.0 µm, and 0.9 µm pixel CIS products.

## Figures and Tables

**Table 1:** Pixel performance comparing Gen1 and Gen2 BSI-stacking technologies (1 µm, 16 MP products).

Parameter	Units	Gen1	Gen2
Array		16 MP	16 MP
Pixel size	µm	1.0	1.0
Full Well Capacity (ADC range)	e^−^	5000	6000
Sens-G (530 nm)	e^−^/(Lux × s)	3150	3600
PRNU (average)	%	0.8	0.8
SNR10 (ΔE = 2.5)	Lux	90	80
Dark current (T = 60 C)	e^−^/s	4	2
Blooming	%	0%	0%
FPN (RT)	[e]	0.5	0.2
Read noise (16x gain)	[e]	2.0	1.4
RTS (>1 mV)	ppm	500	200
